# Beyond anti-inflammatory strategies: addressing immunosuppression with nanomaterials in sepsis treatment

**DOI:** 10.3389/fimmu.2024.1500734

**Published:** 2024-11-18

**Authors:** Zhiyong Wang, Pei Wei

**Affiliations:** Department of Immunology, Zunyi Medical University, Zhuhai, China

**Keywords:** nanomaterials (A), inflammation, immunosuppression, sepsis, bacteria

## Introduction

Sepsis is a life-threatening multi-organ dysfunction syndrome (MODS) associated with dysregulated body responses to infection and is the leading cause of death in intensive care units (1–3). In 2017, a total of 48.9 million cases of sepsis and 11 million sepsis-related deaths were recorded worldwide, the latter accounting for approximately 20% of global deaths (4–6). Despite decades of research, substantial breakthroughs in specific treatments have yet to be achieved. High incidence and mortality rates, coupled with limited non-specific treatment options, render sepsis a significant challenge in the medical field and cause substantial social and economic burdens.

Aggressive early sepsis identification and treatment initiatives are essential to reducing mortality in patients with sepsis. Traditional sepsis treatments primarily focus on infection control (7–9), hemodynamic stability (10–12), and the maintenance of organ function (13–15). However, these traditional treatment strategies often show limited efficacy. Such therapeutic failure may be attributed to various factors, including the development of drug resistance (16, 17), pharmacokinetic alterations induced by sepsis (18), and an inability to address the underlying immune dysregulation (19). Rapidly advancing nanotechnology has unlocked a new stage in sepsis treatment. Nanotechnology is mainly based on the processes of designing, synthesizing, and utilizing nanomaterials (NMs) within the size range of 1–100 nm (20, 21). NMs exhibit unique properties, such as high surface area, tunable dimensions, and the capability to target specific cells or pathogens, enabling them to interact directly with infectious agents or modulate immune responses (22–24). These advantages make NMs particularly effective in pathogen removal, drug delivery, and inflammation reduction, thereby offering promising solutions in areas where traditional therapies fall short (22–24). However, it must be noted that while the prospects of NMs in sepsis treatment are encouraging, their application to date remains mainly focused on antimicrobial and anti-inflammatory responses, somewhat neglecting the complex immunopathological mechanisms of sepsis. From an immunological perspective, sepsis is currently considered to display stages of excessive inflammation and immunosuppression, which dynamically coexist or alternate (25, 26). The occurrence of immunosuppression is increasingly recognized as a key factor in immune dysfunction and sepsis mortality (27, 28). Therefore, we propose that the use of NMs in sepsis treatment should be expanded from an emphasis on anti-inflammatory effects to include a focus on reversing immunosuppression and thus restoring the body’s immune homeostasis.

## Key role of immune dysregulation in the progression of sepsis

To date, there is no gold standard for defining sepsis, as the pathogenesis of this disease involves multiple pathological mechanisms. The gradually increasing understanding of the mechanisms of sepsis has brought with it three successive and significant revisions in the definition of sepsis. Initially, sepsis was defined as a systemic inflammatory response syndrome (SIRS) caused by infection (29, 30). In 2016, new guidelines redefined sepsis as a life-threatening organ dysfunction caused by a dysregulated host response to infection (1). The latest definition of sepsis emphasizes the importance of host immune dysregulation caused by infection, which goes beyond the possibility of direct death from the infection itself (31–33).

The development of immune dysregulation during sepsis is typically divided into two closely related but distinct stages: early systemic inflammatory response and later immunosuppression (25, 26). In the early stages of infection, pathogens enter the body and trigger the activation of the innate immune system through pathogen-associated molecular patterns (PAMPs) (34–36). This response is intended to eliminate pathogens and protect body tissues from further damage. However, in some cases, such as when the infection load is too heavy or the individual’s immune system response is abnormal, the immune response cannot return to homeostasis. The inflammatory response is then further amplified and produces a series of damage-associated molecular patterns (DAMPs) in response to tissue damage and cell death (37, 38). In this process, excessive pro-inflammatory cascades lead to a large accumulation of cytokines (39, 40) and trigger many pathological events, such as complement activation (41, 42), coagulation dysfunction (43, 44), and exhaustion and metabolic reprogramming of immune cells (45–47). These phenomena exacerbate immune dysregulation and cause immunosuppression, which in turn leads to a reduction in infection clearance and makes the individual more susceptible to secondary infections, ultimately worsening the outcome of sepsis.

It is worth noting that these two stages do not strictly appear in chronological order. In fact, they often coexist during the course of sepsis, and which stage predominates may depend on multiple host and pathogen factors (48, 49). Host factors include the individual’s genetic background, age, baseline health status, and comorbidities, while pathogen factors include type, virulence, and load. Growing evidence suggests that immunosuppression is a major cause of sepsis-related mortality, with approximately 70% of clinical deaths in septic patients occurring during this phase (25, 26). Despite significant progress in understanding the immune mechanisms of sepsis, the coexistence and alternation of excessive inflammation and severe immunosuppression add significant complexity to its treatment. For instance, excessive inflammation can promote immunosuppression by inducing immune cell exhaustion and death. Therefore, early anti-inflammatory therapy is essential to mitigate immunosuppression. However, poorly timed or excessive anti-inflammatory treatment can also exacerbate immunosuppression. Hence, achieving a balance between controlling inflammation and preventing immunosuppression is crucial for optimizing therapeutic strategies in sepsis management.

## Current NMs-based treatments for sepsis

NMs have revolutionized the therapeutic approach to sepsis by utilizing a diverse array of materials, including inorganic and organic substances, of both natural and synthetic origins, or even innovative combinations thereof (22–24). This diversity allows for tailored solutions targeting the complex pathophysiology of sepsis. Given the breadth and depth of studies on this topic, it is not feasible to cover all research comprehensively. Therefore, **Table 1** illustrates representative examples of nanoparticles employed in sepsis treatment, categorized by their specific mechanisms of action.

**Table 1:** Mechanisms of anti-septic efficacy of NMs.

Mechanism	Description	Representative NMs	References
Improving drug delivery efficiency	NMs enhance therapeutic delivery in sepsis by protecting drugs from degradation, enabling targeted and stimulus-responsive release, penetrating biological barriers, and ultimately improving efficacy and safety for better treatment outcomes.	Liposome NMs Polymeric NMs Metallic NMs Exosomal-based NMs Biomimetic NMs	(50–61)
Direct pathogen eradication	NMs combat multidrug-resistant bacteria by disrupting cells, inducing oxidative stress, interfering with replication, and enhancing therapies; functionalization improves selectivity, highlighting their potential in treating sepsis.	Metallic NMs Carbonaceous NMs Polymeric NMs Boride NMs Composite NMs	(62–75)
Inhibition of excessive inflammatory response	NMs effectively inhibit excessive inflammatory responses in sepsis by clearing ROS, neutralizing inflammatory mediators from pathogens and the host, and targeting immune cells, thus disrupting inflammation amplification.	Metallic NMs Abiotic hydrogel NMs Magnetic NMs Cerium oxide NMs Biomimetic NMs	(77–84)
Combination therapy	NM-based combination therapies, co-delivering antimicrobial and anti-inflammatory agents, offer a promising approach for sepsis management by simultaneously targeting infection and inflammation.	Metallic NMs Polymeric NMs Magnetic NMs Composite NMs Biomimetic NMs	(85–92)

The anti-septic efficacy of NMs can be broadly categorized based on four key mechanisms. Firstly, NMs serve as highly efficient delivery systems for anti-septic drugs, increasing drug efficacy and reducing systemic side effects. This targeted drug delivery is crucial in sepsis, where rapid and localized intervention is often required. Secondly, certain NMs can directly eradicate pathogens or neutralize toxins produced by these pathogens. This pathogen-specific mechanism of action is particularly valuable in the early stages of sepsis, where the pathogen burden is high. Thirdly, NMs can modulate the host’s immune response to attenuate the overwhelming inflammatory reactions typical of sepsis. By interacting with immune cells or acting as scavengers for inflammatory cytokines, they prevent the progression to severe systemic inflammation and multi-organ failure. Lastly, NMs can be engineered for combination therapy, simultaneously providing antimicrobial and anti-inflammatory effects. This dual functionality ensures a comprehensive approach to sepsis management, addressing both the underlying infection and the detrimental host immune response.

## Improving drug delivery efficiency

NMs provide a multifaceted approach to enhancing the delivery of therapeutic agents, particularly for treating sepsis. Their unique sizes and properties offer significant advantages in various aspects of drug delivery. By encapsulating drugs, they protect these agents from enzymatic degradation and harsh physiological conditions, thereby improving stability and bioavailability until the drugs reach the infection site (50). For instance, liposomal nanoparticles can safeguard antibiotics from premature breakdown in the bloodstream, preserving their potency and allowing for reduced dosing frequencies (51–53). Additionally, NMs can be engineered for intelligent, controlled, and sustained release. They respond to specific environmental stimuli characteristic of infection sites—such as pH changes (54, 55), bacterial enzyme concentrations (56, 57), or reactive oxygen species (ROS) variations (58, 59)—to release their payload precisely when needed. The functionalization of these NMs enables targeted delivery to specific cells or tissues by attaching ligands, antibodies, or peptides to their surfaces. This strategy concentrates therapeutic agents at the site of infection while minimizing adverse effects and reducing the development of drug resistance. Moreover, their ability to overcome biological barriers, such as penetrating bacterial biofilms and accessing intracellular pathogens, facilitates the delivery of antibiotics directly to otherwise inaccessible infection sites (60, 61). Collectively, these capabilities of NMs significantly enhance therapeutic efficacy and safety, ultimately leading to improved treatment outcomes.

## Direct pathogen clearance

Some NMs exhibit inherent antimicrobial properties, offering a promising approach for combating bacterial infections, particularly those caused by multidrug-resistant strains (62, 63). Among these, silver nanoparticles (AgNPs) have been extensively researched for their ability to eradicate a broad spectrum of bacteria through various mechanisms. These include the disruption of bacterial cell walls and membranes, leading to structural damage and increased permeability (64, 65); the promotion of ROS production, which induces oxidative stress and damages vital cellular components (66, 67); and the interference with bacterial DNA and RNA replication, thereby hindering cell proliferation and survival (68, 69). Similarly, gold nanoparticles (AuNPs) and zinc nanoparticles (ZnNPs) also demonstrate intrinsic antimicrobial activity by penetrating bacterial cells and disrupting metabolic processes, resulting in bacterial death (70, 71). Moreover, some NMs serve as catalysts that enhance the effects of external antimicrobial agents or stimuli. For instance, magnetic nanoparticles can be activated under exogenous electromagnetic stimulation, generating localized heating (hyperthermia) or mechanical vibrations that disrupt bacterial cell structures (72, 73). This targeted antimicrobial therapy minimizes damage to surrounding healthy tissues while reducing systemic side effects. Furthermore, surface modification of NMs can significantly improve their interaction with bacterial cells. Functionalizing nanoparticles with specific ligands or antibodies increases their binding affinity to bacterial targets, thereby enhancing selectivity and efficacy (74, 75). Collectively, these advances underscore the potential of NMs as effective agents in the fight against bacterial infections.

## Inhibition of excessive inflammatory response

NMs can also effectively inhibit excessive inflammatory responses by clearing ROS, adsorbing (neutralizing) excessive inflammatory mediators in the body, and targeting immune cells. During the development of sepsis, excessive inflammation causes a large amount of ROS to be produced in cells; these include H_2_O_2_, hydroxyl radicals, and superoxide anions (76). Excessive ROS will cause severe oxidative stress in cells, leading to cell death and consequent release of a large number of DAMPs (37, 38). These are further recognized by pattern recognition receptors (PRRs), forming a vicious cycle of inflammation amplification. Suitably modified NMs, such as tungsten disulfide nanoparticles, mesoporous selenium nanoparticles, and manganese dioxide nanoparticles, demonstrate excellent ROS clearance efficiency and bioavailability, which can effectively inhibit the inflammatory response in sepsis (77, 78). The development of excessive inflammation in sepsis is mediated by various inflammatory mediators, which may derive from pathogens (such as endotoxins) (79) or from the body itself (including free nucleic acids, histones, and pro-inflammatory cytokines) (80). Some NMs, such as chitosan, cationic polymers, and biomimetic nanoparticles, have the capability to scavenge toxins from pathogens or inflammatory mediators produced by the body, thereby inhibiting the progression of excessive inflammation (81, 82). In addition, the use of NMs to target immune cells and block overactive immune responses can also achieve effective control of inflammation (83, 84).

## Combination therapy

The pathophysiology of sepsis is intricate, and relying solely on antimicrobial or anti-inflammatory therapies may be insufficient for effective management. Consequently, numerous researchers have investigated combination therapies that integrate both antimicrobial and anti-inflammatory strategies. One promising approach involves the use of nanomaterials as carriers to co-deliver antibacterial and anti-inflammatory agents, thereby enhancing therapeutic efficacy (85–89). For instance, Zhang et al. developed polymeric nanoparticles capable of efficiently releasing the antibiotic ciprofloxacin and the anti-inflammatory agent TPCA-1, which resulted in a survival rate of up to 90% in septic mice (89). Additionally, treatment strategies can employ inherently antimicrobial nanomaterials as carriers for anti-inflammatory drugs or modify these materials to exhibit dual antimicrobial and anti-inflammatory properties (90–92). Such dual-function nanomaterials offer a versatile platform for addressing the multifaceted nature of sepsis by simultaneously targeting infection and inflammation. These innovative combination therapies not only improve survival outcomes in experimental models but also hold potential for translation into clinical applications, representing a significant advancement in the treatment of sepsis.

## Challenges in the use of NMs for sepsis treatment

NMs are increasingly recognized as a promising medium for antibacterial and anti-inflammatory applications. However, several significant limitations persist in their application in sepsis treatment. The primary issue is that current strategies focus predominantly on suppressing hyperinflammation, with insufficient attention being paid to modulating the immunosuppressive phase. Several points should be noted in this regard.

Firstly, sepsis, as a heterogeneous disease syndrome, does not simply follow a strict sequence of systemic inflammatory response and immunosuppressive phases. In many cases, these two phases may overlap, with dominance depending on various factors related to the host and pathogen (25, 26). Given the complexity of sepsis pathogenesis, distinguishing these two phases to determine the optimal timing for NM treatment is a challenging task. Implementing aggressive anti-inflammatory treatment at an inappropriate time could conceivably exacerbate a sepsis condition already in the immunosuppressive phase. Such misdirected treatment may suppress essential immune functions, heightening the risk of secondary infections and worsening patient outcomes. Therefore, a nuanced understanding of the patient’s immunological status is crucial for the effective application of NM therapies.

Secondly, it is well known that the surge of inflammatory molecules is a necessary condition for the immune system to combat invading pathogens (93, 94). Although NMs possess potent anti-inflammatory properties, excessive suppression of the inflammatory response could lead to earlier and more intense immunosuppression, making the host more susceptible to secondary infections. In fact, in some drug trials, the mortality rate of sepsis patients was shown to increase due to excessive suppression of the inflammatory response (95). Similarly, while intracellular ROS can promote inflammation and potentially cause various cellular dysfunctions, an appropriate level of ROS generation is beneficial for pathogen clearance (96, 97). In contrast, large-scale clinical trials have demonstrated that supplementation with classical antioxidants (such as vitamins C and E) has limited efficacy and may even increase the risk of sepsis (11, 98). Similarly, while the strategy of controlling sepsis through the adsorption of inflammatory mediators or endotoxins by NMs seems theoretically feasible, currently available treatments have not demonstrated a reduction in mortality rates in clinical practice (11). Some literature suggests that suppressing pro-inflammatory cytokines during infection does not provide protective effects against sepsis (99–101). Notably, several trials have reported an increased mortality rate in patients with Gram-positive bacterial infections following anti-endotoxin treatment (102).

Thirdly, the application of NMs in sepsis therapy presents a paradoxical challenge due to their potential to invoke significant pro-inflammatory responses. The introduction of NMs into biological systems can activate immune cells, leading to the excessive release of pro-inflammatory cytokines and chemokines (103–105). This hyperinflammatory state not only results in direct tissue damage but also exacerbates the subsequent immunosuppressive phase characteristic of sepsis. Such overactivation depletes immune resources, compromising the body’s ability to combat secondary infections and leading to more severe immune dysfunction (103–105). Additionally, the physicochemical attributes of NMs—such as size, shape, surface charge, and composition—critically influence their biocompatibility and immunogenicity. Accumulation in vital organs may provoke cytotoxic effects and oxidative stress, further impairing organ function (106–108). Therefore, a comprehensive understanding of these factors is essential to mitigate adverse effects.

Fourthly, it is crucial to recognize that the development of sepsis is often associated with dysbiosis of the host’s microbiota. Clinical studies have demonstrated that patients experiencing microbiota imbalance, particularly following hospital interventions like antibiotic treatments, have a significantly increased risk of developing sepsis and septic shock within 90 days (109, 110). Dysbiosis disrupts the delicate equilibrium of commensal microorganisms that play a vital role in immune system modulation. This disruption can impair gut barrier function, leading to translocation of pathogens into the bloodstream and triggering systemic inflammation (111, 112). Moreover, dysbiosis may promote immunosuppression by altering the maturation and function of immune cells, rendering the host more susceptible to infections (113). Although some NMs possess broad-spectrum antibacterial properties, their use could inadvertently exacerbate host dysbiosis. The non-selective elimination of beneficial microbiota might further compromise the immune system, negatively impacting sepsis treatment outcomes. Therefore, while designing nanomaterial-based therapies, it is imperative to consider their effects on the host microbiota.

Fifthly, it is imperative to consider that the efficacy of NMs in sepsis treatment may be significantly modulated by individual host differences. As previously noted, the progression of the inflammatory response and subsequent immunosuppressive phases in sepsis is profoundly influenced by host-specific factors. Notably, the trajectory of the immunosuppressive phase exhibits substantial heterogeneity among different patient populations, including adults, the elderly, infants, and individuals with underlying conditions such as diabetes or cancer (114–120). These comorbidities and age-related changes can alter immune function, affecting both the innate and adaptive immune responses. Consequently, the therapeutic effectiveness and safety profile of NMs may vary widely across these groups. Most preclinical studies investigating the anti-sepsis effects of NMs employ healthy adult animal models, which do not adequately represent the clinical diversity encountered in practice (121–123). This discrepancy inevitably leads to a significant gap between experimental outcomes and real-world applicability. To bridge this gap, it is essential to incorporate diverse animal models that mimic the immunological states of different patient populations. Moreover, personalized medicine strategies should be considered to tailor NM treatments according to individual host characteristics, thereby optimizing therapeutic efficacy and minimizing adverse effects.

Lastly, it must be acknowledged that environmental pollution, both extracorporeal and intracorporeal, could significantly impact NMs. Before entering the host body, numerous potential pollutants present in the environment can easily adsorb onto the surface of NMs. For example, LPS is a heat-resistant and widely distributed potential pollutant. Current methods for its detection are easily subject to interference by various factors, making it difficult to exclude in nanomaterial experiments (124, 125). The mechanisms by which LPS modulates immune responses are complex and could exacerbate inflammation or induce immunosuppression, thereby masking the true effects of NMs. Moreover, once NMs are introduced into the host, they can rapidly interact with a myriad of biomolecules—including proteins, nucleic acids, lipids, and metabolites—to form a “protein corona” (126). This dynamic biointerface can alter the physicochemical properties and immunogenicity of NMs, potentially promoting immune imbalance within the host. Therefore, it is crucial to consider both environmental and biological factors that may influence NM behavior, and to develop rigorous purification and characterization protocols to mitigate these effects in clinical applications.

## NMs for dynamic immunomodulation in sepsis: a paradigm shift in treatment

**Figure 1:**
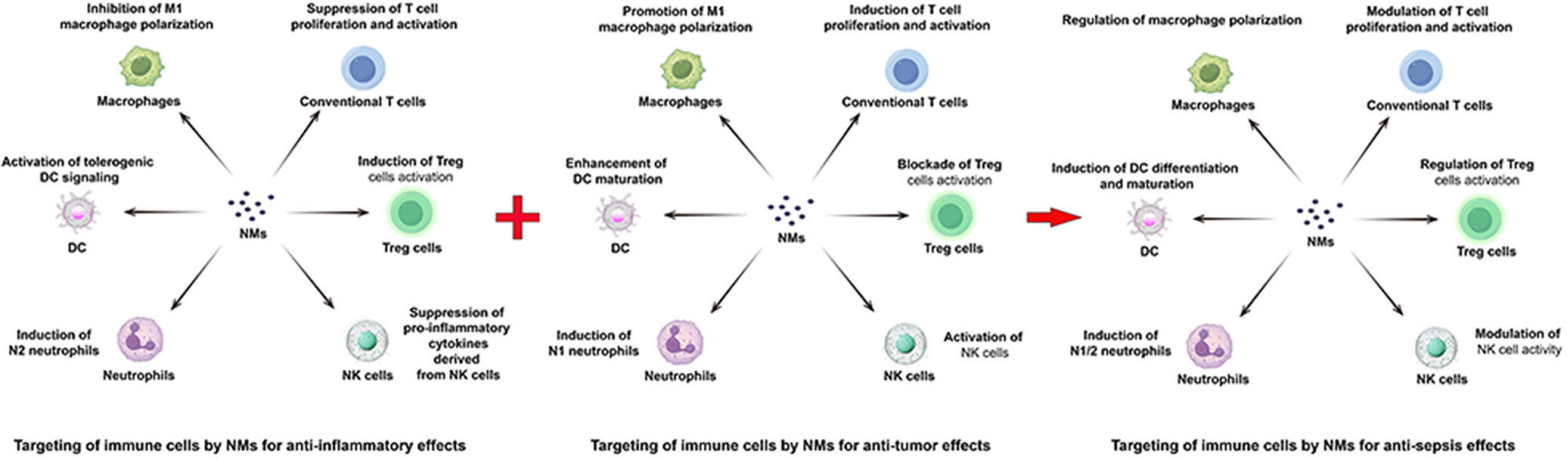
Mechanistic representation of NM-mediated targeted immunomodulation in sepsis. NMs leverage their combined anti-inflammatory and immune-activating properties, mirroring strategies employed in tumor, to address the biphasic nature of sepsis. In the early inflammatory phase, NMs suppress inflammation. As the disease progresses into the immunosuppressive phase, NMs promote immune reconstitution. This dynamic interplay, depicted centrally, represents a novel therapeutic paradigm for sepsis treatment, integrating the established anti-inflammatory and immune-reactivating capabilities of NMs for targeted immune cell modulation.

The pathogenesis of sepsis is characterized by a complex and dynamic interplay between inflammation and immunosuppression, a duality that renders simplistic anti-bacterial and anti-inflammatory approaches insufficient. Effective therapeutic intervention necessitates a nuanced strategy focused on precise immune modulation, tailored to the evolving phases of the disease. During the predominantly inflammatory phase, anti-inflammatory strategies should be prioritized. Conversely, during the subsequent immunosuppressive phase, effective immune reconstitution becomes paramount. This transition highlights the need for adaptive therapies that can dynamically respond to the shifting immunological landscape. The immunosuppressive phase of sepsis is marked by widespread immune cell dysfunction, leading to systemic immune paralysis. This includes a shift from M1 to M2 macrophage polarization, T cell exhaustion, increased regulatory T cell (Treg) counts, elevated immature neutrophil populations, enhanced dendritic cell apoptosis, and impaired natural killer (NK) cell function (45–47). While current NM-based therapies for sepsis largely focus on anti-bacterial and anti-inflammatory effects, their substantial potential for immune reconstitution remains largely unexplored. The ability of NMs to specifically target and modulate diverse immune cell subsets, a capability extensively demonstrated in the field of oncology, suggests a promising therapeutic strategy for sepsis treatment. Integrating the established immune-activating properties of NMs, well-characterized in anti-cancer applications (127–130), with their inherent anti-inflammatory capabilities, presents a transformative therapeutic paradigm for sepsis management. As depicted in **Figure 1**, this strategy envisions a dynamic therapeutic shift between anti-inflammatory and immune-reconstitutive modalities, adapting to the evolving needs of the patient throughout the course of the disease. However, realizing this vision requires the development of robust, real-time monitoring tools capable of accurately assessing the individual patient’s disease stage and immunological profile. Furthermore, comprehensive safety assessments, meticulously evaluating potential toxicities and accounting for inter-individual variability based on age, comorbidities, and genetic background, are critical for the successful clinical translation of such NMs. This rigorous approach will be essential to ensure both efficacy and safety in this complex disease setting.

## Conclusion and prospects

In summary, despite the tremendous potential of NMs in sepsis treatment, current application strategies primarily focus on suppressing hyperinflammation. This limited approach may neglect the modulation of the immunosuppressive phase of sepsis, potentially exacerbating the disease course. To overcome the existing limitations of NMs in sepsis treatment, we anticipate the following trends in future developments. Firstly, given the significant differences among sepsis patients in terms of infection source, degree and stage of immune response, age, gender, and comorbidities, the importance of personalized treatment should be emphasized. The design and application of future NM treatment strategies need to consider each patient’s specific status and needs, achieving a “precision medicine” approach. Secondly, the disease course of sepsis typically includes an excessive inflammatory response phase followed by an immunosuppressive phase. Therefore, an ideal treatment strategy should be able to adaptively respond to these two phases, exerting anti-inflammatory effects during hyperinflammation and activating immune responses to restore immune balance during immunosuppression. Future research on NMs may focus on developing nanotherapeutic systems with this “smart” response capability. Lastly, the precise detection of molecular biomarkers by NMs constitutes a unique advantage of these materials and a crucial step towards personalized treatment. By integrating diagnosis, treatment, and monitoring into a single nano-platform, it is possible to track a patient’s immunopathological status in real time and adjust treatment strategies promptly. This integrated diagnostic-therapeutic nano-system not only provides more precise and efficient treatment plans for patients but also helps evaluate treatment effects, which is crucial for controlling the development and prognosis of sepsis. Overall, NMs offer many new perspectives and tools for sepsis treatment, warranting further exploration and development.
